# Fast and accurate object detector for autonomous driving based on improved YOLOv5

**DOI:** 10.1038/s41598-023-36868-w

**Published:** 2023-06-15

**Authors:** Xiang Jia, Ying Tong, Hongming Qiao, Man Li, Jiangang Tong, Baoling Liang

**Affiliations:** China Telecom Corporation Limited Beijing Research Institute, Beijing, China

**Keywords:** Electrical and electronic engineering, Computer science

## Abstract

Autonomous driving is an important branch of artificial intelligence, and real-time and accurate object detection is key to ensuring the safe and stable operation of autonomous vehicles. To this end, this paper proposes a fast and accurate object detector for autonomous driving based on improved YOLOv5. First, the YOLOv5 algorithm is improved by using structural re-parameterization (Rep), enhancing the accuracy and speed of the model through training-inference decoupling. Additionally, the neural architecture search method is introduced to cut redundant branches in the multi-branch re-parameterization module during the training phase, which ameliorates the training efficiency and accuracy. Finally, a small object detection layer is added to the network and the coordinate attention mechanism is added to all detection layers to improve the recognition rate of the model for small vehicles and pedestrians. The experimental results show that the detection accuracy of the proposed method on the KITTI dataset reaches 96.1%, and the FPS reaches 202, which is superior to many current mainstream algorithms and effectively improves the accuracy and real-time performance of unmanned driving object detection.

## Introduction

In recent years, there has been a significant increase in the number of motor vehicles, which has greatly facilitated people's travel. However, this increase has resulted in increasingly crowded traffic conditions and a rise in the frequency of traffic accidents, which has posed a significant challenge to safe travel. In the face of an increasingly complex traffic environment, individuals are often required to rely on their own experience to choose a suitable travel route, and to deal with various emergencies that may arise on the road. Even experienced drivers are not immune to encountering unpredictable hazards.

With the development of computer technologies such as big data and artificial intelligence, technical means such as smart cities and automatic driving have provided new solutions to alleviate traffic pressure and traffic safety problems^1,2^. Whether it is a smart city or autonomous driving, it is necessary to analyze the traffic scene to obtain useful information, that is, to perceive the external environment. Computer vision technology is the most convenient and fast technical means to perceive the external environment at this stage, and object detection is the most basic and critical task in computer vision. Object detection recognizes the category and position of targets in the image, which provides detailed basic environmental information for scene analysis in computer vision. Therefore, the detection of targets in traffic scenes has become an indispensable research direction^3,4^.

When the object detection algorithm is applied in the traffic scene, the algorithm has high requirements. The algorithm not only needs to have a high recognition accuracy but also demands to meet the requirements of the real scene^5^. Most of the previous research on object detection focuses on how to improve the detection accuracy of the algorithm and further optimize the existing network by increasing the number of network layers. Although the detection accuracy of the model can be improved to a certain extent, the large model makes it difficult for the algorithm to run on devices with low computing power and the detection speed is undesirably low. In most traffic scenarios, devices are used outdoors, especially in the field of autonomous driving, where the hardware devices used to run algorithms cannot have large computing power^6,7^.

In the field of transportation, the development of both autonomous driving technology and smart cities is increasingly inseparable from smart terminal devices. In the process of realizing autonomous driving, most of the terminal devices used in the car are limited by space and environmental factors such as power supply, and most of them are devices with low power consumption and small computing resources. And in the field of autonomous driving, not only the object detection algorithm but also other perception algorithms and driving control algorithms are required to meet the needs of autonomous driving^8,9^. The development of smart cities needs to obtain accurate traffic environment information through cameras and other external devices in order to make timely adjustments to traffic conditions. However, more sensors have higher performance requirements for data transmission and central processors, and using smart terminal devices to return processed information can increase the processing speed and reliability of the system. In this case, although the existing larger object detection algorithms have better performance in terms of accuracy, they obviously cannot be applied to most traffic scenarios on account of their poor detection speed performance. Therefore, it’s a tricky challenge to make the object detection algorithm as fast as possible without affecting the accuracy performance of the algorithm, so that the object detection algorithm can be transplanted to the terminal application on the vehicles and achieve a real-time autonomous driving object detection^10,11^.

In summary, accuracy and speed are two significant indicators in the field of automatic driving object detection. The high accuracy enables the object detection algorithm to more precisely locate and identify vehicles or pedestrians ahead, while fast speed makes the model acquire the changes of external objects more quickly, thereby assisting the control system to operate more reasonably to ensure the safety of the occupants in the vehicle. Therefore, designing an object detection algorithm with high precision and fast speed is critical for object detection of unmanned driving.

For ordinary deep learning methods of autonomous driving object detection, accuracy and speed are two indicators that are difficult to balance. This paper proposes a fast and accurate object detector based on improved YOLOv5 algorithm, which has achieved double improvement in detection accuracy and speed. The main contributions are summarized as follows.

- The YOLOv5 algorithm is used as the baseline algorithm, the structural re-parameterization (Rep) module is introduced for improvement, and the accuracy and speed of the model are improved through training-inference decoupling.
- The neural architecture search (NAS) method is applied to the structural re-parameterization module, and redundant branches in the multi-branch module are automatically cut off, which improves the efficiency and accuracy of model training.
- For the problem of low detection accuracy in small vehicle and pedestrian targets, a small object detection layer is added and coordinate attention (CA) is inserted into all detection layers to improve the model's recognition accuracy for small and illegible objects.

The rest of this article is organized as follows. Section “Related work” describes the related work of unmanned driving object detection. Section “Methodology” introduces YOLOv5, Rep, NAS, small object detection layer, CA, and improved YOLOv5 comprehensively. Section “Experiment and results” presents the experimental results as well as some detailed discussion. Section “Conclusions” summarizes the work of this article.

## Related work

### Vehicle detection

Vehicle detection is the most important and common recognition scenario in autonomous driving scenarios. Accurate and rapid recognition of other vehicles on the road is of great significance to ensure the safety of the occupants and avoid vehicle collisions. Dong et al.^12^ introduced C3Ghost and Ghost modules into the YOLOv5 neck network to reduce the computational cost, adopted convolutional block attention module to select the information critical to the vehicle detection task, and utilized CIoU_Loss to accelerate the bounding box regression rate. Chen et al.^13^ proposed an improved SSD algorithm for quick vehicle detection in traffic scenes, which selects MobileNetV2 as the backbone and utilizes the deconvolution module to construct a bottom–top feature fusion architecture. Aiming at designing an algorithm managing the speed and accuracy of the detector in real-time vehicle detection, Zarei et al.^14^ proposed Fast-Yolo-Rec algorithm. They combined a new Yolo-based detection network improved by semantic attention mechanism and LSTM-based position prediction networks for the specified trajectory and vehicle position prediction. Mittal et al.^15^ presented a hybrid model combining Faster R-CNN and YOLO. They used majority voting classifier and compared it with its base estimators on several vehicle detection datasets, verifying that the proposed approach can effectively enhance road traffic management.

### Pedestrian detection

Pedestrian detection is also a critical topic in autonomous driving. Especially in places with a large flow of people and dense crowds, it is necessary to accurately identify pedestrians to ensure road safety. HSU et al.^16^ introduced a segmentation strategy which can split pedestrians into several images. And the strategy further performed multiresolution adaptive fusion on the output of all images to generate the final pedestrian recognition result. And then they verified the effectiveness of the proposed model by conducting an extensive evaluation of several pedestrian detection data sets. To address the problem that large pedestrian detection networks cannot adapt to edge computing scenarios due to the high computational cost and slow detection speed, Liu et al.^17^ proposed a pedestrian detection and recognition model MobileNet-YOLO based on the YoLov4-tiny object detection framework, which adopts the lightweight MobileNetv3 backbone and CBAM attention mechanism. Wang et al.^18^ proposed a small object detection method based on image super-resolution and enhanced the speed and accuracy of tiny object detection, which introduces a feature texture transfer module, dense blocks, and balance loss function to YOLOv4. Shao et al.^19^ presented AIR-YOLOv3 for aerial infrared pedestrian detection, which combines network pruning and the YOLOv3 method, significantly decreasing the computational cost and improving the detection speed.

### Lane line detection

Realizing efficient lane detection is one of the important components of the road environment perception module of unmanned vehicles. Lane line detection can prevent vehicles from driving out of the road track, and its accuracy also affects the safety of unmanned vehicles. To increase the accuracy of lane detection in complex scenarios, Zhang et al.^20^ suggested an adaptive lane feature learning algorithm that automatically learns the features of lane lines in complex scenarios. They constructed a two-stage network based on the YOLOv3, presented a way for the automatic generation of the lane label images in simple scenarios, and used an adaptive edge detection method based on the Canny operator to relocate the lane recognized by the first-stage algorithm. To improve lane detection performance in a complicated environment, Haris et al.^21^ proposed an approach combining visual information and spatial distribution, which improves the grid density of the object detection algorithm YOLOv3 and presented a new lane line prediction model BGRU-Lane. To solve the problems of low detection accuracy and poor real-time performance of traditional methods, Huu et al.^22^ advised a lane and object detection algorithm, which improves the quality of the distorting image caused by the camera, implements the sliding window to determine pixels of each lane, and utilizes YOLO algorithm to identify lanes and obstacles (Table 1).

**Table 1:** Relevant characteristics of related works.

Topic	Authors	Method	Characteristic
Vehicle detection	Dong et al	Ghost-YOLOv5	Introducing C3Ghost and Ghost modules into the YOLOv5 neck network
	Chen et al	Improved SSD	Selecting MobileNetV2 as the backbone of SSD
	Zarei et al	Fast-Yolo-Rec	Combining YOLO improved by semantic attention mechanism and LSTM
	Mittal et al	Faster R-CNN and YOLO	Combining Faster R-CNN and YOLO, and using majority voting classifier
Pedestrian detection	HSU et al	A segmentation strategy	Splitting pedestrians into several images and performing multiresolution adaptive fusion
	Liu et al	MobileNet-YOLO	Improving YOLOv4-tiny by MobileNetv3 backbone and CBAM attention mechanism
	Wang et al	Improved YOLOv4	Introducing feature texture transfer module, dense blocks, and balance loss to YOLOv4
	Shao et al	AIR-YOLOv3	Combining network pruning and the YOLOv3 method
Lane Line detection	Zhang et al	Adaptive lane feature learning algorithm	Automatically learning the features of lane lines in complex scenarios
	Haris et al	YOLOv3 and BGRU-Lane	Improving the grid density of YOLOv3 and presenting a lane line prediction model BGRU-Lane
	Huu et al	Sliding window and YOLO	Implementing sliding window to determine pixels of each lane and using YOLO to identify lane

## Methodology

### YOLOv5 algorithm

**Figure 1:**
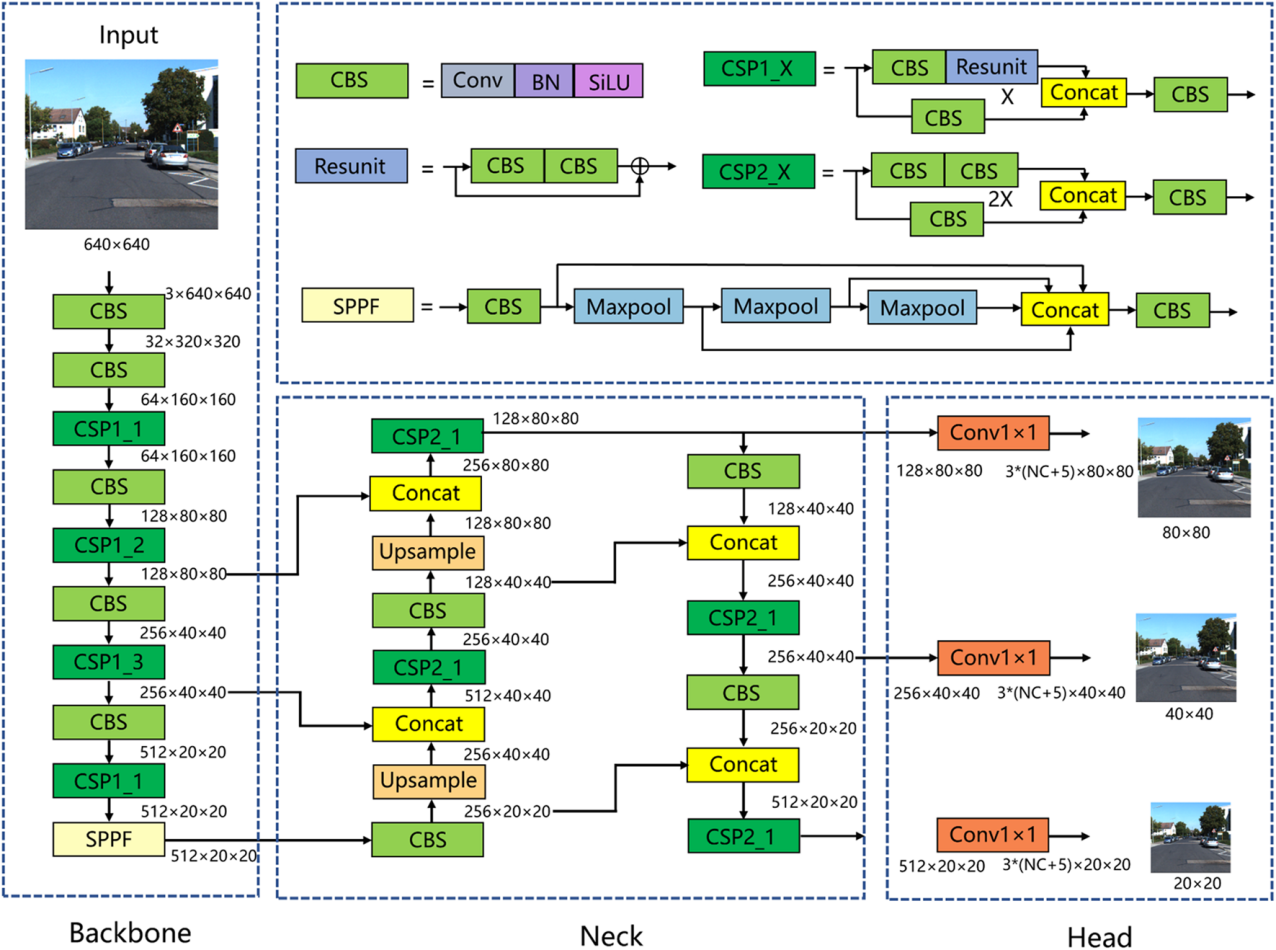
Network architecture of YOLOv5.

YOLOv5 is currently one of the most mainstream single-stage object detection algorithms. The YOLOv5 algorithm consists of three modules: CSP-DarkNet backbone, FPN + PAN neck, and prediction head. As shown in Fig. 1, a picture with a size of 3 × 640 × 640 is input into the network. In the backbone network part, the CBS layer is used for downsampling, and the CSP module is used for feature extraction. After 5 times of downsampling, the size of the feature map becomes 512 × 20 × 20. Finally, an SPPF module is connected to realize the fusion of feature maps of different receptive fields. In the neck network part, the feature map first passes through a dimensionality reduction path, and then through a dimensionality enhancement path. Feature maps with sizes of 512 × 20 × 20, 256 × 40 × 40, and 128 × 80 × 80 are fully fused through two paths. In the head network part, feature maps of three sizes enter the detection head and then pass through a 1 × 1 convolutional layer. The size remains unchanged, and the number of channels becomes 3 × (NC + 5), where 3 represents three types of anchor boxes with different aspect ratios, NC represents the number of categories, and 5 represents 4 parameters used to indicate the position of the anchor frame plus 1 anchor frame foreground probability.

### Structural re-parameterization

Structural re-parameterization^23,24^ is a method that uses training-inference decoupling to achieve a model with both high accuracy in the training phase and high speed in the inference phase. Specifically, a multi-branch structure is first constructed in the training phase, and after training, the multi-branch structure is fused into a one-way structure for model inference and deployment. In today's actual autonomous driving scenarios, the inference model is often deployed on the edge AI chip, and the captured pictures are detected on the edge device in real time. Therefore, the model needs to have fast inference speed under the premise of ensuring accuracy and structural re-parameterization can satisfy this condition very well.

Structural re-parameterization consists of the following basic fusion modules:

1. A Conv-BN layer is fused into a Conv layer: $F_{j,:,:,:}^{{}}$ and $F_{j,:,:,:}^{{\prime }}$ represent the weight of the $j$ th convolutional kernel in the convolutional layer before and after fusion respectively, and $b_{j}^{{\prime }}$ represents the $j$ th bias in the fused convolutional layer. Then the fused convolutional layer weight and bias can be expressed as:

1 $$ F_{j,:,:,:}^{{\prime }} = \frac{{\gamma_{j} }}{{\sigma_{j} }}F_{j,:,:,:} $$

2 $$ b_{j}^{{\prime }} = - \frac{{\mu_{j} \gamma_{j} }}{{\sigma_{j} }} + \beta_{j} $$

where $\mu ,\sigma ,\gamma ,\beta$ are the cumulative mean, standard deviation, learned scaling factor, and bias factor of the BN layer, respectively.

2. Multiple parallel Convs are fused into one Conv layer: all convolutional layers are filled to the same size, and the residual branch can be regarded as a 1 × 1 convolutional layer whose parameters are unit matrices. $F^{{\prime }}$ and $b^{{\prime }}$ denote the weight and bias of the convolutional layer after fusion, respectively, and $F^{i}$ and $b^{i}$ denote the weight and bias of the convolutional layer on the parallel branch after filling, respectively. Then the weight and bias of the convolutional layer after fusion can be expressed as:

3 $$ F^{^{\prime}} = F^{1} + F^{2} + \cdots + F^{N} $$

4 $$ b^{^{\prime}} = b^{1} + b^{2} + \cdots + b^{N} $$

where $N$ is the number of parallel branches.

3. 1 × 1 Conv and $k \times k$ Conv in series are fused into one $k \times k$ Conv: $F^{1}$ and $F^{2}$ represent the weights of the 1 × 1 convolutional layer and the $k \times k$ convolutional layer, respectively; $b^{1}$ and $b^{2}$ represent the bias of the 1 × 1 convolutional layer and the $k \times k$ convolutional layer, respectively; $F^{{\prime }}$ and $b^{{\prime }}$ represent the weight and bias of the fused $k \times k$ convolutional layer, which can be expressed as:

5 $$ F^{{\prime }} = F^{2} * TRANS(F^{1} ) $$

6 $$ b_{j}^{{\prime }} = \sum\limits_{d = 1}^{D} {\sum\limits_{u = 1}^{K} {\sum\limits_{v = 1}^{K} {b_{d}^{1} F_{j,d,u,v}^{2} + b^{2} } } } $$

where $*$ represents the convolution operation and $TRANS()$ represents the transposition of the tensor on the 0 and 1 dimensions.

**Figure 2:**
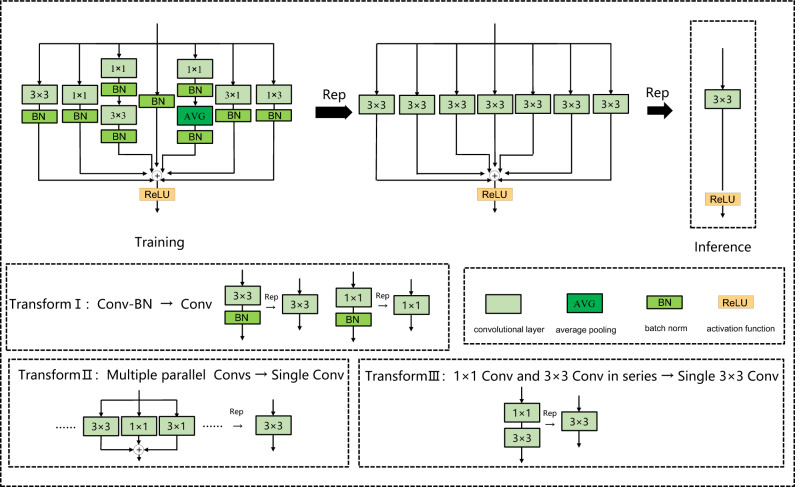
Structural reparameterization module.

In this paper, seven branches are used to build a structural re-parameterization module. As shown in Fig. 2, in the training phase, the model contains a 3 × 3 convolutional layer branch, a 1 × 1 convolutional layer branch, a 1 × 1–3 × 3 branch, an identity branch, a 1 × 1-AVG branch, a 3 × 1 convolutional layer branch, and a 1 × 3 convolutional layer branch. Each branch includes one or two batch normalization layers. After the model training is completed, the seven branches can be fused through the above-mentioned model fusion methods I, II, and III, and converted into a one-way structure for inference.

### Neural architecture search

**Figure 3:**
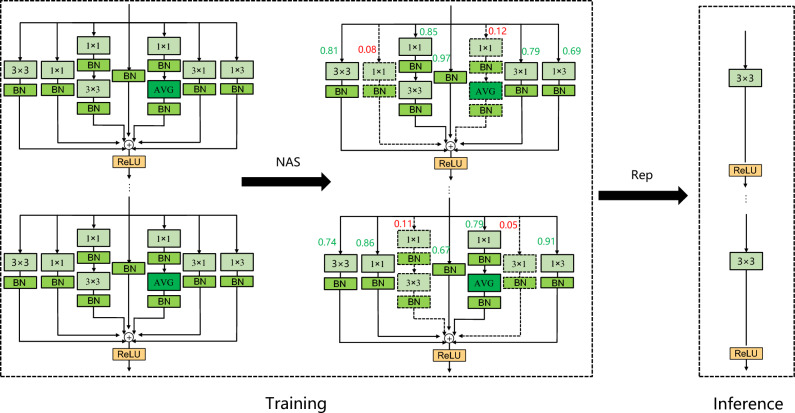
RepNAS module.

Neural architecture search is a current research hotspot in the field of computer vision, which uses strategies such as reinforcement learning, evolutionary algorithms, and gradient methods to automatically search for the optimal structure of the network. Inspired by Zhang et al.^25^, this paper combines the NAS technology with the structural re-parameterization module described in Section “Structural re-parameterization”, and designs the RepNAS module, as shown in Fig. 3. RepNAS automatically cuts some redundant branches by judging the importance of different branches in multiple branches, so as to achieve the effect of reducing model training time and memory, and improving model accuracy. The specific steps are as follows:

First, judge the importance of each branch according to formula (7):

7 $$ Z_{i,j} = \frac{1}{{1 + exp((\alpha_{i,j} + \zeta_{i,j} )/\lambda_{i,j} )}} $$

where $Z_{i,j}$, $\alpha_{i,j}$, $\zeta_{i,j}$, and $\lambda_{i,j}$ represent the importance of the branch in the structure re-parameterization module, structural parameters, sampling noise, and temperature coefficient, respectively.

Second, calculate whether each branch is activated according to formula (8):

8 $$ \left\{ {\begin{array}{*{20}c} {\mathop {lim}\limits_{{\lambda_{i,j \to 0 + } }} Z_{i,j} = 0,} & {if\,R_{i,j} < 0\,and\,rank(R_{i,j} ) < s} \\ {\mathop {lim}\limits_{{\lambda_{i,j \to 0 - } }} Z_{i,j} = 0,} & {if\,R_{i,j} > 0\,and\,rank(R_{i,j} ) < s} \\ {\mathop {lim}\limits_{{\lambda_{i,j \to 0 - } }} Z_{i,j} = 1,} & {if\,R_{i,j} < 0\,and\,rank(R_{i,j} ) > s} \\ {\mathop {lim}\limits_{{\lambda_{i,j \to 0 + } }} Z_{i,j} = 1,} & {if\,R_{i,j} > 0\,and\,rank(R_{i,j} ) > s} \\ \end{array} } \right. $$

where $R_{i,j} = \alpha_{i,j} + \zeta_{i,j}$ and $rank(R_{i,j} )$ represents the importance ranking of the $j$ th branch in all roads in the $i$ th structure re-parameterization module, and whether to activate the branch is decided by setting the threshold.

Finally, the relevant weights are updated by the gradient of the weight parameter through the loss function and $\alpha_{i,j}$ is updated by the gradient calculated by Eq. (9).

9 $$ \frac{\partial L}{{\partial \alpha_{i,j} }} = \frac{\partial L}{{\partial x_{i} }}O_{i,j}^{T} f(\alpha_{i,j} )(1 - f(\alpha_{i,j} ))/\lambda_{i,j} $$

### Small object detection layer

The detection head of the YOLOv5 network contains a total of three detection layers, and the scales are 80 × 80, 40 × 40, and 20 × 20, respectively. Among them, the 80 × 80 detection layer has the smallest area per square, and the position information is more accurate, so it is more suitable for detecting small objects. Similarly, the 40 × 40 detection layer is suitable for detecting medium objects, while the 20 × 20 detection layer is more suitable for detecting large objects. In vehicle detection or pedestrian detection, many objects account for a small proportion of the original image. In order to improve the detection accuracy of the detection algorithm for small targets, we add a 160 × 160 detection layer for accurate positioning and recognition of smaller vehicles or pedestrians.

### Coordinate attention

Aiming at the problem that the recognition accuracy is not high due to the small proportion of certain vehicles and pedestrians in the input image, this paper introduces the coordinate attention mechanism. It can encode horizontal and vertical position information into the channel attention mechanism so that the network can better focus on the target position information.

**Figure 4:**
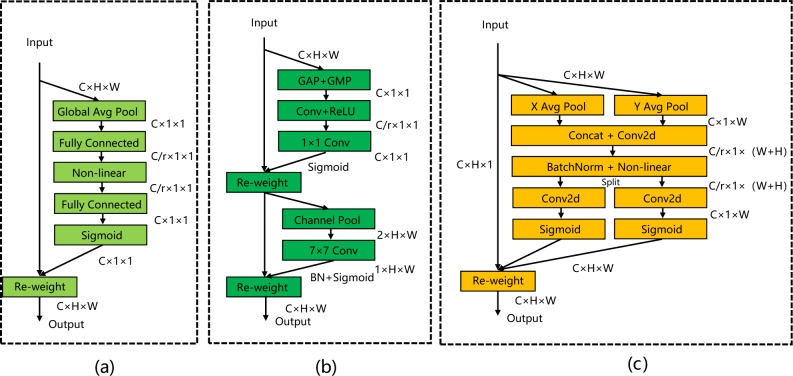
Diagrams of three attention mechanisms. (**a**) SE. (**b**) CBAM. (**c**) CA.

The classic SE^26^ attention mechanism is shown in Fig. 4a, which only considers the information between channels and ignores the position information. CBAM^27^ improves SE, as shown in Fig. 4b, which uses convolution to extract positional attention information after reducing the number of feature map channels. However, convolution can only extract local relations, and it is difficult to pay attention to long-distance information. CA^28^, as shown in Fig. 4c, is able to encode horizontal and vertical position information into channel attention, and simultaneously captures inter-channel information and direction-dependent position information. It can improve the model's ability to perceive the target position, thereby achieving a more accurate location and identification of cars and pedestrians. The generation steps of CA's attention mechanism are as follows:

First, the coordinate information is embedded, as shown in formula (10)–(12):

10 $$ z_{c} = \frac{1}{H \times W}\sum\limits_{i = 1}^{H} {\sum\limits_{j = 1}^{W} {x_{c} (i,j)} } $$

11 $$ z_{c}^{h} (h) = \frac{1}{W}\sum\limits_{0 \le i \le W} {x_{c} (h,i)} $$

12 $$ z_{c}^{w} (w) = \frac{1}{H}\sum\limits_{0 \le j \le H} {x_{c} (j,w)} $$

where $x_{c}$ is the input of the given $c$ th channel, $z_{c}^{h} (h)$ represents the output of the $c$ th channel whose height is $h$, and $z_{c}^{w} (w)$ represents the output of the $c$ th channel whose width is $w$.

Secondly, the generation of coordinate attention is carried out, as shown in formula (13)–(15):

13 $$ {\text{f}} = \delta (F_{1} (\left[ {z^{h} ,z^{w} } \right])) $$

14 $$ g^{h} = \sigma (F_{h} ({\text{f}}^{h} )) $$

15 $$ g^{w} = \sigma (F_{w} ({\text{f}}^{w} )) $$

where [ , ] represents the concat operation, $\delta$ represents the activation function, and f represents the intermediate feature map that encodes spatial information in the horizontal and vertical directions.

Finally, the output of the coordinate attention mechanism can be written as:

16 $$ y_{c} (i,j) = x_{c} (i,j) \times g_{c}^{h} (i) \times g_{c}^{w} (j) $$

### Improved YOLOv5 network

**Figure 5:**
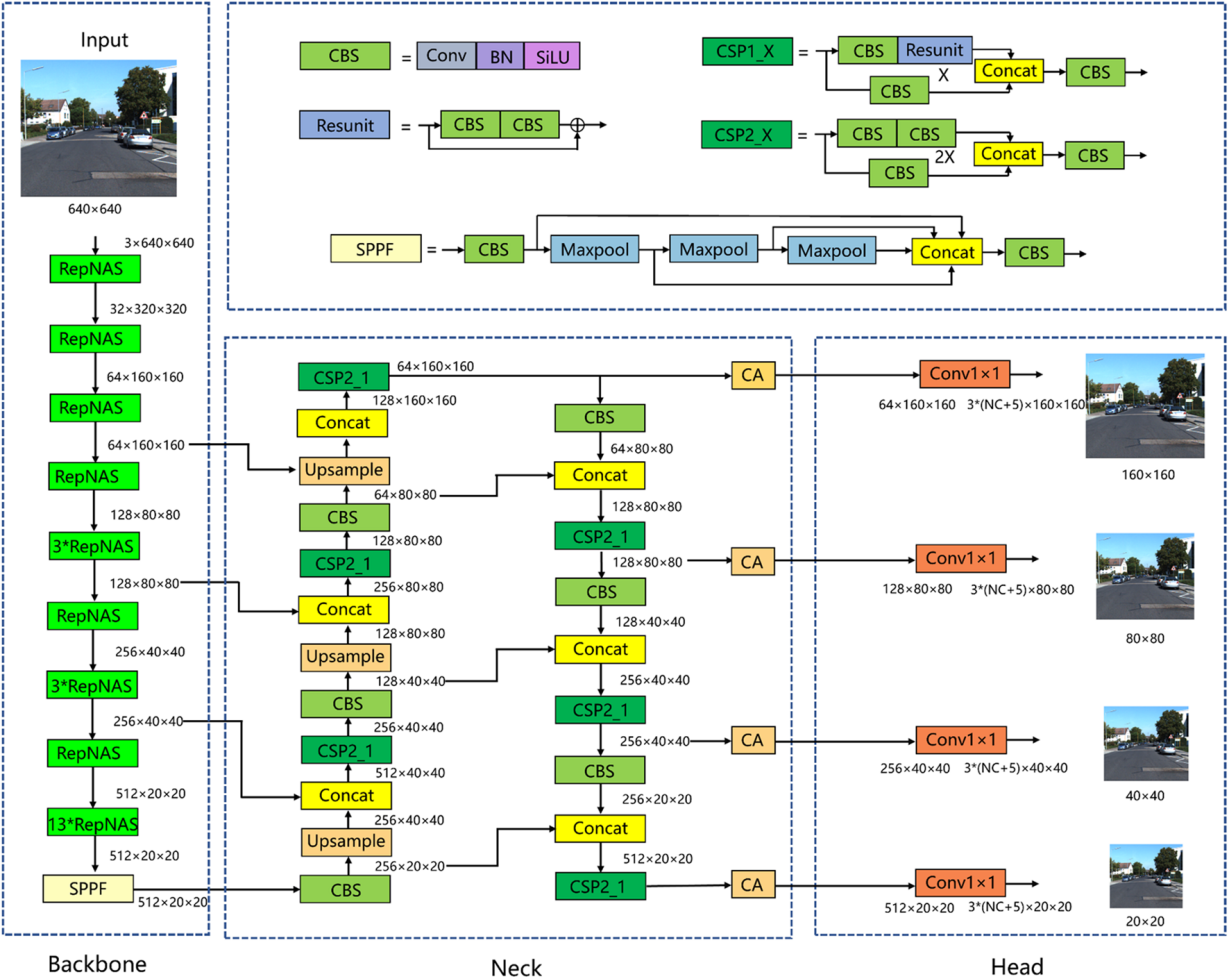
Improved YOLOv5 algorithm.

The network structure diagram of the improved YOLOv5 algorithm is shown in Fig. 5. First, the modules in the backbone network are all changed to RepNAS modules, and the number of downsampled RepNAS modules is 1, while the numbers of non-downsampled RepNAS modules are 1, 3, 3, and 13, respectively. As described in sections “Structural re-parameterization” and “Neural architecture search”, during the training phase, the non-downsampled RepNAS module contains 7 branches, while the downsampled RepNAS module contains six branches because the input and output feature map sizes of the downsampled RepNAS module are different, and the identity branch does not exist. NAS judges the importance of different branches of each module during the training phase, and constantly cuts out unimportant branches to reduce model redundancy and improve model training accuracy and training efficiency. Rep realizes the simplification of the RepNAS module structure through the equivalent transformation of the parameters after the training. That is, all the RepNAS modules in the backbone network are converted into 3 × 3 convolutional layers so that the backbone network becomes a VGG-style architecture during the inference phase, making the inference speed significantly fast and the high accuracy of model training maintained. Second, a small object feature detection layer with a size of 64 × 160 × 160 is added. As shown in Fig. 5, in the neck network, the 160 × 160 feature map utilizes the shallow information in the backbone network for feature fusion. The shallow network feature map has a higher resolution and contains more small object information, so adding a detection layer with a size of 160 × 160 can achieve better positioning and recognition of small targets. Finally, each detection layer is preceded by a CA attention mechanism. The reason for adding CA before the detection layer is that the feature map before the detection layer has been fully extracted and fused, and the semantic information is complete. Therefore, adding the attention mechanism here can make the model apply more attention to the semantically rich channels.

### Informed consent

Informed consent has been obtained from all individual participants to publish the information and images in an online open access publication.

## Experiment and results

### Dataset and experimental environment

We conduct experiments on the most widely used KITTI dataset in the field of autonomous driving. The KITTI training set is marked with 7481 images, including road scenes such as rural areas, urban areas, and highways. There are at most 15 vehicles in each image, and the target has different degrees of occlusion and truncation. The data set contains a total of 8 categories: Car, Van, Truck, Tram, Pedestrian, Person(sitting), Cyclist, and Misc. Among them, we merge Person into Pedestrian category, select Car, Van, Truck, and Cyclist in addition, and take out the five types of objects for the training and testing. All data are randomly divided into training set, validation set, and test set in a ratio of 8:1:1. In order to enhance the training effect of the model on small targets, the Mosaic data enhancement method is used to randomly select 4 images in the training set, and stitch them into a new image by random scaling, cropping, flipping, and colour gamut changes.

The experimental environment is Ubuntu 21.04, Pytorch 1.8.0, CPU model 11th GenIntel (R) Core (TM) i5-11,400@2.60 GHz, GPU model NVIDIA GeForce RTX 3070, memory 16G, CUDA 11.2, CUDNN 7.6, Python3.8. The initial learning rate is 0.01, cosine annealing decay is used, the final learning rate is 0.001, the epoch is set to 300, and the batch size is set to 8.

### Evaluation metrics

We use precision P (Precision), recall rate R (Recall), accuracy mAP (Mean Average Precision) and FPS (Frames per Second) of the model as the relevant indicators to evaluate the performance of the model. The specific calculation formula is as follows:

17 $$ p = \frac{TP}{{TP + FP}} $$

18 $$ R = \frac{TP}{{TP + FN}} $$

where $TP$ means that the positive sample is predicted to be positive, $FP$ means that the positive sample is predicted to be negative, and $FN$ means that the negative sample is predicted to be positive.

19 $$ AP = \int_{0}^{1} {P(R)dR} $$

where $\int_{0}^{1} {P(R)dR}$ represents the area enclosed by the $P - R$ curve and the coordinate axis obtained by setting different confidence levels under the premise that the recall rate is the abscissa and the precision is the ordinate.

20 $$ mAP = \frac{{\sum\limits_{i = 1}^{N} {AP_{i} } }}{N} \times 100\% $$

where $N$ is the number of categories and $AP_{i}$ is $AP$ of the *i*th category.$mAP@0.5$ indicates the model accuracy when the intersection ratio threshold is set to 0.5.

### Ablation experiment

In order to evaluate the impact of each improved component on the model's performance, an ablation experiment is conducted on the improved YOLOv5 algorithm, and the results are shown in Table 2.

**Table 2:** Ablation experiment results of improved YOLOv5.

Model	Rep	NAS	Small object detection layer	CA	P/%	R/%	mAP@0.5/%	FPS
YOLOv5					89.7	93.5	92.9	155
A	√				90.3	94.8	94.1	263
B	√	√			91.1	95.9	94.7	263
C	√	√	√		92.4	96.9	95.8	213
ours	√	√	√	√	92.9	97.7	96.1	202

The original YOLOv5 algorithm achieves a high accuracy of 92.9% and a fast inference speed of 155FPS, indicating a good balance between accuracy and efficiency for object detection.

The addition of the structural re-parameterization module improves the model’s accuracy by 1.2% and increases its FPS by 108. This highlights the effectiveness of the structural re-parameterization method, which utilizes 7 branches during training to improve model fitting accuracy and fuses them into a 3 × 3 convolutional layer during inference to achieve fast detection speed. The analysis from Section “Structural re-parameterization” support this finding.

Incorporating NAS into the model improves its accuracy by 0.6%, and the FPS remains the same. NAS is applied to structural re-parameterization modules, and unimportant branches in the multi-branch are cut off during the training process. On the one hand, it effectively prevents model overfitting, and on the other hand, it also eliminates the negative impact of redundant branches on model accuracy, thereby further improving the accuracy of the model. Furthermore, it reduces model size and memory usage, thereby accelerating the training process. Section “Neural architecture search” provides additional demonstration to support these claims.

The addition of the small object detection layer results in a 1.1% increase in model accuracy but decreases FPS by 50. This is due to the increased parameter quantity and computational demands of the model, resulting in slower detection speed, as explained in Section “Small object detection layer”. However, the accuracy gain outweighs the speed decrease, making this a valuable improvement to the overall performance of the model.

Introducing the CA attention mechanism improves model accuracy by 0.3%, albeit with a slight decrease in FPS. The CA attention mechanism enhances the network detection layer's ability to focus on the target's location information, thus improving accuracy. However, it also introduces additional computational demands, causing a minor drop in FPS, as discussed in Section “Coordinate attention”.

Overall, the incorporation of these four methods into the YOLOv5 model improves its accuracy by 3.2% and increases its FPS by 47, resulting in more accurate and efficient object detection for unmanned vehicles.

### Test of different branches in rep module

In order to investigate the impact of adding different branches to the structural re-parameterization module on the model and the effect of NAS under different branch combinations, relevant experiments are carried out, and the results are presented in Table 3.

**Table 3:** Test results of different branches in Rep module.

3 × 3	1 × 1	Identity	1 × 1–3 × 3	1 × 1-AVG	1 × 3	3 × 1	Without NAS		With NAS	
							mAP@0.5/%	FPS	mAP@0.5/%	FPS
							92.9	155	92.9	155
**√**							87.6	263	87.6	263
**√**	√						88.1	263	88.1	263
**√**	√	√					93.4	263	93.5	263
**√**	√	√	√	√			93.9	263	94.2	263
**√**	√	√	√	√	**√**	√	94.1	263	94.7	263

Initially, when only 3 × 3 branch is used in the structural re-parameterization module, the network architecture resembles VGG, and the accuracy is only 87.6%. In this case, the use of structural re-parameterization is futile, since the same architecture is used for both training and inference phases. Additionally, this indicates that directly applying the VGG-style architecture to the backbone network of YOLOv5 for training would significantly reduce accuracy.

The addition of a 1 × 1 branch results in a small 0.5% increase in accuracy. This minor improvement suggests that increasing the number of branches is beneficial for enhancing the model's accuracy.

The introduction of the residual branch leads to a substantial improvement in accuracy, with a 5.3% increase in mAP@0.5. This highlights the significance of the residual structure in the structural re-parameterization module. The residual structure resolves the problem of gradient disappearance and explosion during deep neural network training, effectively preventing network degradation, which is crucial for improving model accuracy.

The inclusion of 1 × 1–3 × 3 and 1 × 1-AVG branches brings the accuracy rise by 0.5%. However, after 1 × 3 and 3 × 1 branches are added, the accuracy only increases by 0.2%. Under this circumstance, the fitting ability of the model is close to saturation, and continuing to increase branches has little significance for improving the model accuracy.

After NAS is introduced, when the number of branches is small, it does not improve the accuracy of the model much. As the number of branches increases, the improvement effect of NAS on model accuracy is gradually obvious. This is because the more branches, the greater the redundancy of the branches, and NAS effectively improves model accuracy by cutting redundant branches to eliminate its negative impact on model accuracy.

### Comparison of different attention mechanisms

In order to compare the impact of different attention modules on model performance, this paper has added three attention mechanisms to the detection layer on the basis of structural re-parameterization, NAS, and small object detection layer improvements on YOLOv5 and carried out related experiments, as shown in Table 4. The impact of SE and CBAM on the model is similar, the accuracy is increased by 0.1%, and the FPS is decreased by 6. The improvement effect of CA on the model accuracy is obviously better than that of SE and CBAM, and the model accuracy is increased by 0.3%. Combined with Section “Small object detection layer”, it can be seen that CA more comprehensively considers the position information of the model, so it is more effective for the vehicle and pedestrian detection task in this paper.

**Table 4:** Comparisons of different attention mechanisms.

Attention Mechanism	P/%	R/%	mAP@0.5/%	FPS
/	92.4	96.9	95.8	213
SE	92.1	97.2	95.9	207
CBAM	92.7	96.8	95.9	207
CA	92.8	97.2	96.1	202

### Comparison of different object detection models

Performances of several mainstream object detection algorithms are compared, and the results are shown in Table 5. Among the several algorithms other than our proposed method, the Faster-RCNN^29^, Cascade R-CNN^30^, YOLOv5, and YOLOv7^31^ algorithms have relatively high detection accuracy. Faster-RCNN and Cascade R-CNN are two-stage detection algorithms, so they have high recognition accuracy at the expense of detection speed, and their FPS is much lower than that of several other one-stage models. YOLOv5 uses mosaic enhancement and improved CSP-DarkNet to achieve better accuracy, and the FPS is also higher, surpassing RetinaNet^32^, SSD^33^, and YOLOv3^34^ in terms of accuracy and speed. YOLOv7 applies re-parameterization to YOLOv5 and achieves better model performance. Our proposed has the highest accuracy and the fastest speed among all the algorithms in Table 5, which fully proves the superiority of our proposed method.

**Table 5:** Comparisons of different object detectors.

Methods	P/%	R/%	mAP@0.5/%	FPS
Faster- RCNN	89.1	92.8	91.9	78
Cascade R-CNN	88.5	91.9	91.2	72
RetinaNet	85.2	88.7	87.2	134
SSD	85.9	88.8	87.5	123
YOLOv3	89.2	90.9	90.8	108
YOLOv5	89.7	93.5	92.9	155
YOLOv7	92.3	97.1	95.2	132
Ours	92.9	97.7	96.1	202

### Detection result comparison

**Figure 6:**
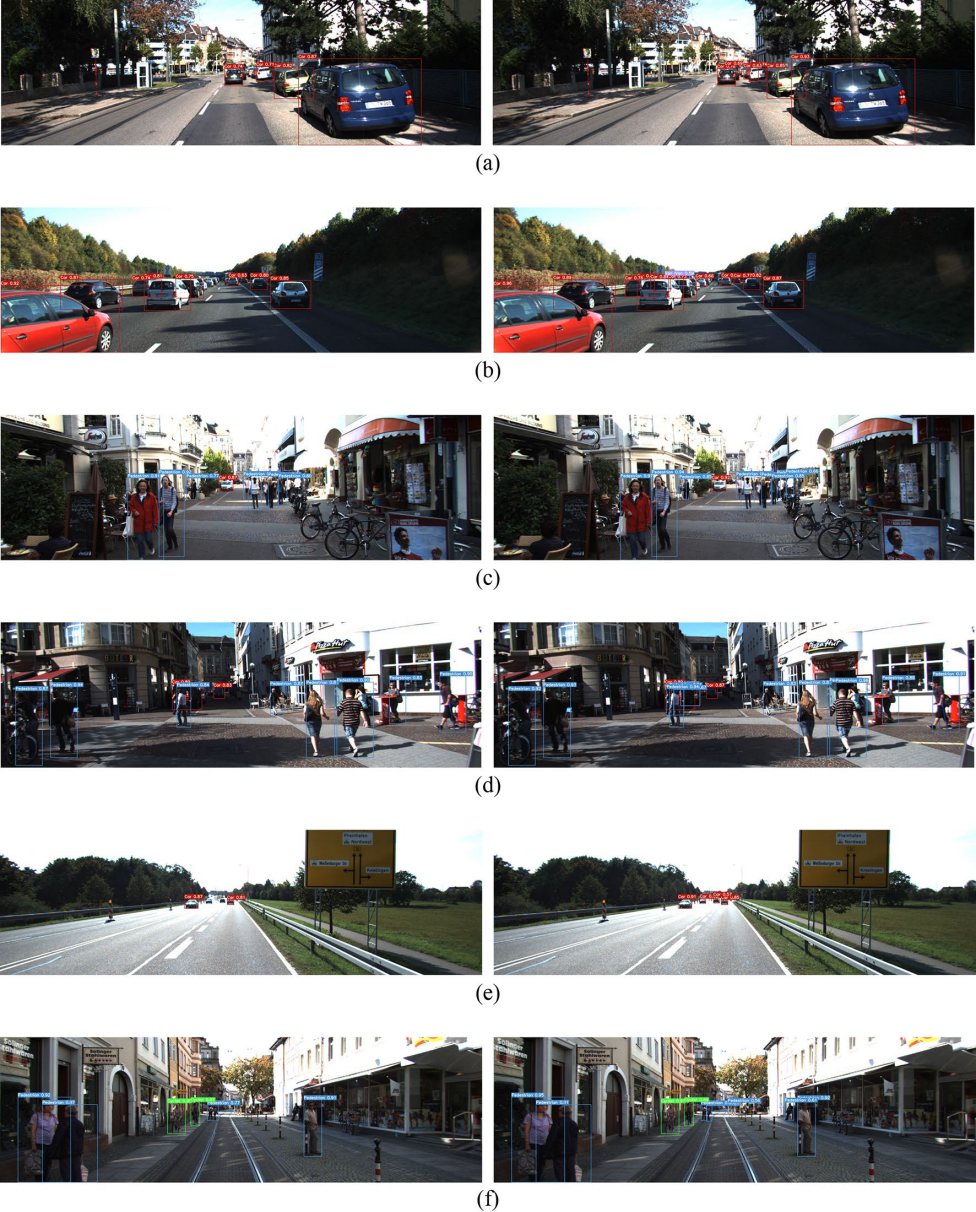
Detection result comparison (left: YOLOv5; right: improved YOLOv5).

An example of the detection results of the YOLOv5 algorithm and the improved YOLOv5 on the test set is shown in Fig. 6. For large targets, both algorithms can identify accurately, and the recognition confidence of our proposed method is generally higher than that of YOLOv5, indicating that the improved YOLOv5 has a better recognition effect in terms of foreground probability than YOLOv5. In some scenes with dense targets, as shown in Fig. 6a–d, due to the serious overlapping and occlusion of vehicles or pedestrians, the YOLOv5 algorithm has missed detection, but the improved YOLOv5 still accurately recognizes the target. In addition, for some distant vehicles or pedestrians, which occupy a small area in the picture, as shown in Fig. 6e, f, the improved YOLOv5 is significantly better than YOLOv5 for the recognition of small targets. Therefore, the detection result example also verifies the effectiveness of our proposed method.

## Conclusions

In order to improve the detection accuracy and speed of vehicles and pedestrians in the autonomous driving scenario, this paper proposes a fast and accurate object detector based on improved YOLOv5. First, by introducing the structural re-parameterization module into the YOLOv5 backbone network, the model achieves improvement in both accuracy and speed through training-inference decoupling, with the mAP@0.5 increased by 1.2%, and the FPS enhanced by 108. In addition, the NAS technology is used to ameliorate the structural re-parameterization module, which accelerates the model training efficiency and increases the accuracy by 0.6%. NAS boosts the accuracy better as the number of branches in the structural re-parameterization module increases. Then, the model recognition rate for small targets is further improved by adding a small object detection layer, with mAP@0.5 increased by 1.1%. Finally, CA attention mechanism is utilized for achieving better attention to different channels and object locations, which exceeds SE and CBAM, and brings a 0.3% improvement in accuracy. Overall, the accuracy of our proposed method is 3.2% higher than that of YOLOv5, and the FPS is increased by 47, which realizes a more quick and precise unmanned driving object detection.

## Data Availability

The data provided in this study can be obtained from the corresponding author X.J.
